# Redesigning the Q_A_ binding site of Photosystem II allows reduction of exogenous quinones

**DOI:** 10.1038/ncomms15274

**Published:** 2017-05-03

**Authors:** Han-Yi Fu, Daniel Picot, Yves Choquet, Guillaume Longatte, Adnan Sayegh, Jérôme Delacotte, Manon Guille-Collignon, Frédéric Lemaître, Fabrice Rappaport, Francis-André Wollman

**Affiliations:** 1 Laboratoire de Physiologie Membranaire et Moléculaire du Chloroplaste, UMR 7141, Institut de Biologie Physico-Chimique, CNRS/Université Pierre et Marie Curie, Paris 75005, France; 2 Laboratoire de Biologie Physico-Chimique des Protéines Membranaires, UMR 7099, Institut de Biologie Physico-Chimique, CNRS/Université Paris Diderot, Paris 7, Paris 75005, France; 3 Ecole Normale Supérieure-PSL Research University, Département de Chimie, Sorbonne Universités, UPMC Univ. Paris 06, CNRS UMR 8640 PASTEUR, 24, rue Lhomond, Paris 75005, France

## Abstract

Strategies to harness photosynthesis from living organisms to generate electrical power have long been considered, yet efficiency remains low. Here, we aimed to reroute photosynthetic electron flow in photosynthetic organisms without compromising their phototrophic properties. We show that 2,6-dimethyl-*p*-benzoquinone (DMBQ) can be used as an electron mediator to assess the efficiency of mutations designed to engineer a novel electron donation pathway downstream of the primary electron acceptor Q_A_ of Photosystem (PS) II in the green alga *Chlamydomonas reinhardtii*. Through the use of structural prediction studies and a screen of site-directed PSII mutants we show that modifying the environment of the Q_A_ site increases the reduction rate of DMBQ. Truncating the C-terminus of the PsbT subunit protruding in the stroma provides evidence that shortening the distance between Q_A_ and DMBQ leads to sustained electron transfer to DMBQ, as confirmed by chronoamperometry, consistent with a bypass of the natural Q_A_°^−^ to Q_B_ pathway.

## Abstract (web summary)

Devices that harness electron flow from photosynthetic organisms generally compromise host photosynthesis. Here, the authors show that, by redesigning the Q_A_ site of Photosystem II, it is possible to reroute electrons to an exogenous quinone while maintaining endogenous photosynthetic electron transfer in a green alga.

The prospect of biophotovoltaics whereby organisms performing oxygenic photosynthesis are exploited to generate electric power emerged over the last decade^1^,^2^,^3^. It consists in utilizing the remarkable characteristics of photosynthetic reaction centres to generate electrical power. The potentialities of natural photosystems rely on their very high maximum quantum efficiency^4^,^5^. Yet, extracting electrons from these photosystems to generate a photocurrent requires the establishment of sustained flux that will be determined by the intrinsic rate constant of light-independent reactions. Currently, these kinetic limitations are such that the yield of biophotovoltaics is still far lower than the theoretical limit of conversion efficiency^2^.

To circumvent these limitations, attempts were made to optimize the electrical connectivity between isolated photosystems and the electrode that eventually collects the reducing equivalents they produce under illumination. For instance, the ZnO-binding peptide was used to facilitate electron transfer from PSI to ZnO anodes when this peptide was linked to the PsaE subunit, which allows tight docking of PSI to the anode^6^. Alternatively, single amino acid substitutions were made within photosystems to change their surface charge density and favour electron transfer to the electrode. As an example, the D1-K238 mutation on the stromal surface of PSII led to higher photocurrent in *Synechocystis*^7^,^8^.

Other approaches consisted in using native photosynthetic membranes in order to alleviate the negative impact of biochemical purification of photosystems on the cost/efficiency ratio of the final biophotovoltaic device. The most common redox mediators used to extract electrons are quinones, which have been successfully employed with PSII-enriched membranes^9^, thylakoid membranes^10^,^11^ or intact cells of cyanobacteria^12^,^13^,^14^. Chloro-substituted benzoquinones were initially taken as effective electron acceptors of PSII^15^,^16^,^17^. Later, it was postulated that several derivatives of benzoquinone undergo reduction by displacement of plastoquinone from their binding site in the Q_B_ binding pocket of PSII, and/or by direct oxidation of the plastoquinone pool in the thylakoid membranes^18^,^19^,^20^. The direct electron transfer from immobile semiquinone Q_A_°^−^ to exogenous quinones was also reported^21^, suggesting that further modification of the structure of the Q_A_ binding pocket may increase the reduction rate of exogenous quinones.

Irrespective of the velocity with which quinones, native or exogenous, accept electrons from PSII, the kinetic limiting step of photosynthesis is located downstream of PSII. Therefore, PSII does not turnover at its maximal rate under saturating illumination conditions. Yet, under such conditions, the PSII primary electron acceptor accumulates in its reduced state, which promotes charge recombination. This decreases the overall conversion efficiency of charge separation but also provides a potent source of reactive oxygen species that damages the photosynthetic machinery. The cost of the ensuing damage/repair cycles actually is a main source of energetic loss in photosynthesis and accounts for its relatively poor yield^22^.

We explored the possibility of designing an additional electron transfer pathway branched in parallel with the endogenous pathway, which would drive electrons out of PSII, when the semiquinone Q_A_°^−^ accumulates. By decreasing the concentration of Q_A_°^−^, such design would decrease the probability of photodamage and thus increase both the overall energetic yield of the biophotovoltaic device and its lifespan. If engineered to preserve phototrophy via regular electron transfer downstream of PSII, the design would allow using viable microalgae, exposed to saturating light intensity, to collect, with an electrode, electrons from a long-lived semi-quinone at the Q_A_ site. Here we modify the environment of the Q_A_ site in the green alga *Chlamydomonas reinhardtii* and screen site-directed PSII mutants for their ability to enhance electron transfer to exogenous quinones. Our results demonstrate that shortening the distance between Q_A_ and exogenous quinones increases the reduction rate of exogenous quinones and thus decreases electron accumulation at the Q_A_ site, consistent with our design aimed at rerouting electrons from Q_A_ to exogenous quinone.

## Results

### Contrasting efficiency of quinones as PSII electron acceptors

**Figure 1:**
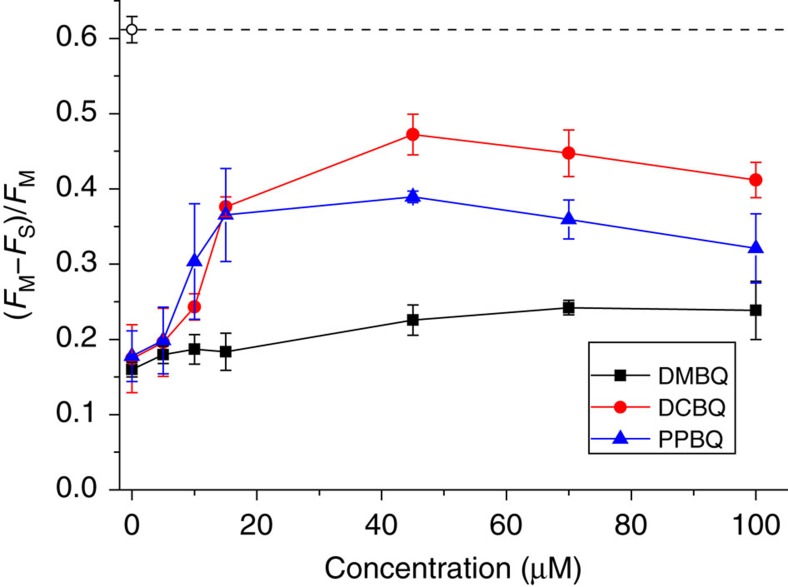
Efficiency of electrons extraction for three quinone derivatives in the Δ*petA* strain lacking the cytochrome *b*_6_*f* complex. The steady-state fluorescence yield (*F*_S_) was measured after ∼2.6 s of illumination at 26 μmol photons m^−2^ s^−1^, while the maximum fluorescence (*F*_M_) was measured after a saturating light pulse (250 ms). Variations in (*F*_M_−*F*_S_)/*F*_M_ values, related to the PSII photochemical efficiency, were used to estimate the efficiency of electrons extraction (mean±s.d.; *n*=3). The restoration of PSII photochemical efficiency in the Δ*petA* strain by the exogenous quinones indicates their ability to act as electron acceptors. The empty circle and the dashed line show the (*F*_M_−*F*_S_)/*F*_M_ value in the WT reference strain in the absence of exogenous quinones under the same light condition (mean±s.d.; *n*=10).

To assess the relative efficiency of various quinones to accept electrons from PSII, we used a mutant strain of *C. reinhardtii* lacking cytochrome *b*_6_*f* (Δ*petA*), a protein complex which acts as a quinol:plastocyanin oxidoreductase. In this strain, illumination progressively reduces the plastoquinone pool and consequently Q_A_°^−^, leading to an increase of the fluorescence yield. Thus, the ability of exogenous quinones to accept electron from PSII, that is, to restore the electron flow downstream of PSII, is witnessed by a decrease in the steady-state fluorescence level reached under continuous illumination with sub-saturating light. This can be quantitatively assessed by the ratio (*F*_M_−*F*_S_)/*F*_M_, where *F*_M_ is the maximum fluorescence yield probed by a saturating multi-turnover light pulse and *F*_S_ is the fluorescence yield under continuous illumination probed before the pulse. Figure 1 demonstrates that addition of any of the three quinone derivatives induced an increase of the (*F*_M_−*F*_S_)/*F*_M_ value, although 2,6-dimethyl-*p*-benzoquinone (DMBQ) was markedly less efficient than 2,6-dichloro-*p*-benzoquinone (DCBQ) and phenyl-*p*-benzoquinone (PPBQ).

### Electron-acceptor sites of exogenous quinones

**Figure 2:**
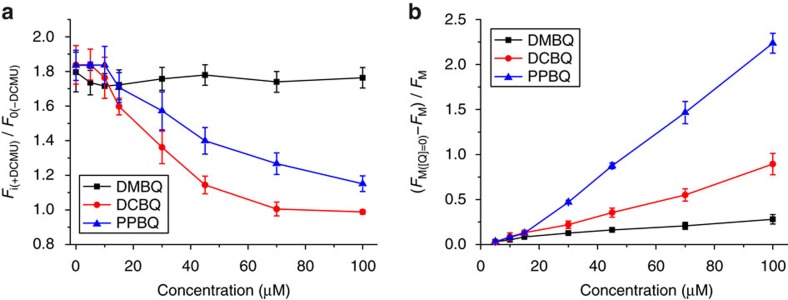
Ability of three quinone derivatives to deplete electrons from Q_B_°^−^ and/or PQ^2−^ in the WT strain using chlorophyll fluorescence analysis. (**a**) Effect of quinone derivatives on the ratio of initial fluorescence in the presence of 10 μM DCMU (*F*_i_) to that in its absence (*F*_0_) (mean±s.d.; *n*=3). Addition of DCMU induces a rise of the initial fluorescence level that reflects the residual electron accumulation in the Q_B_ site or the plastoquinone pool in the dark. (**b**) Stern–Volmer plot of the maximum fluorescence yield (*F*_M_) showing the fluorescence quenching effect of exogenous quinones (mean±s.d.; *n*=3). *F*_M([Q]=0)_ represents the *F*_M_ value in the absence of quinones.

The following question was at which location in the electron transport chain the exogenous quinones could accept electrons downstream of Q_A_°^−^. In the absence of exogenous quinones, addition of 3-(3,4-dichlorophenyl)-1,1-dimethylurea (DCMU) induces a rise of the initial fluorescence yield (from *F*_0_ in its absence to *F*_i_ in its presence) due to the inability of DCMU to enter the Q_B_ binding pocket when it is occupied by Q_B_°^−^, as happens in about 50% of the PSII centres in algae kept in darkness^23^. Owing to the equilibrium between the Q_A_Q_B_°^−^ and Q_A_°^−^Q_B_ states, PSII centres in the former state transiently reverse to the Q_A_°^−^Q_B_ state, allowing DCMU to compete with Q_B_ for occupying the ‘Q_B_ pocket', thus closing all centres originally in the Q_B_°^−^ state in darkness^24^. The rise of the initial fluorescence yield observed upon addition of DCMU is thus a measure of the fraction of PSII centres having Q_B_ in its semiquinone state. We measured the DCMU-induced rise from *F*_0_ to *F*_i_ upon addition of exogenous quinones as a marker of their ability to oxidize Q_B_. Whereas DCBQ and PPBQ almost completely cancelled the fluorescence rise observed upon addition of DCMU, the *F*_i_/*F*_0_ ratio was only marginally affected by the addition of up to 100 μM DMBQ (Fig. 2a). Thus, DMBQ poorly interacts with the Q_B_ binding site and the downstream plastoquinone pool with which Q_B_ is at equilibrium. Since the three quinones are benzoquinones that do not differ greatly in terms of reduction potential of their relevant redox couples, all the three quinones can readily accept electrons based on the midpoint potential data^25^. We therefore interpret this poor interaction, as being due to the mediocre partitioning of DMBQ into the thylakoid membranes. This conclusion was further substantiated by the Stern–Volmer plot of *F*_M_ for the three quinone derivatives that showed that DCBQ and PPBQ—but not DMBQ—are efficient fluorescence quenchers (Fig. 2b). To interact directly with the excited states of protein-bound chlorophylls and decrease their lifetime by a mechanism that would involve the transient formation of a charge-transfer state^26^, these quinones must be embedded within thylakoid membranes. Altogether with the results in Fig. 1, we conclude that DMBQ has little interaction with the plastoquinone pool or the Q_B_ binding site of PSII. We thus selected this quinone to assess our engineering strategy aimed at designing an additional electron transfer pathway from Q_A_°^−^ to the stroma.

### Extraction efficiency using absorbance changes of P_700_

**Figure 3:**
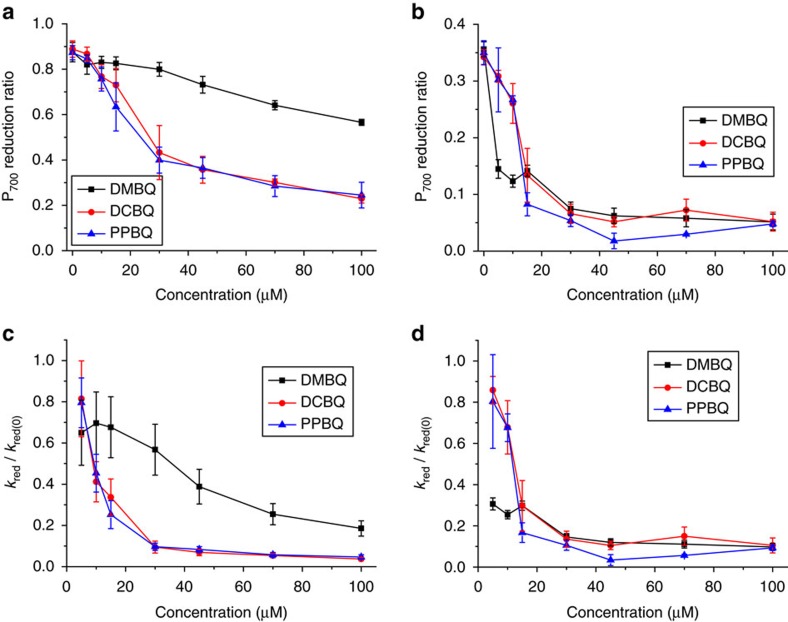
Efficiency of electron extraction for three quinone derivatives in the WT strain using light-induced absorbance changes of P_700_. (**a**) P_700_ reduction ratio as a function of exogenous quinone concentration (mean±s.d.; *n*=4). P_700_ redox states were estimated from the absorbance changes at 705−730 nm. The P_700_ reduction ratio was calculated as 1 minus the oxidation level reached after a 10 s illumination (26 μmol photons m^−2^ s^−1^) over the level of full P_700_^+^ oxidation after a saturating light pulse. Decreases in the P_700_ reduction ratio indicate a reduced electron flux towards P_700_^+^ in the presence of exogenous quinones. (**b**) P_700_ reduction ratio as a function of exogenous quinone concentration in the presence of 10 μM DCMU (mean±s.d.; *n*=3). (**c**,**d**) P_700_ reduction rate (*k*_red_), normalized to the rate measured in the absence of quinones (*k*_red(0)_), as a function of exogenous quinone concentration, derived from the data of (**a**,**b**), respectively. The normalized *k*_red_/*k*_red(0)_ ratio is particularly useful to compare strains or conditions that have different basal levels of P_700_ reduction ratio, as seen here after DCMU treatment.

To assess the effect of exogenous quinones in the WT strain, which was further used as a recipient strain for site-directed mutagenesis, we used the reduction rate of P_700_, the primary chlorophyll donor of PSI, as a measure of the ability of exogenous quinones to divert electrons from the regular photosynthetic electron flux towards P_700_, and hence to decrease its relative reduction state under steady-state illumination of non-saturating light. Such decrease became significant when using DCBQ and PPBQ at concentrations of about 20 μM, while, in agreement with the results above, the effect of DMBQ on the reduction state of P_700_ was far more limited (Fig. 3a; see Methods and Supplementary Fig. 1a for the estimation of the P_700_ reduction ratio). The same experiments were also conducted in the presence of the PSII inhibitor DCMU (10 μM), to assess whether the addition of exogenous quinones impacts cyclic electron flow (Fig. 3b). Addition of exogenous quinones also led to a decrease of the P_700_ reduction ratio. It is of note that, in contrast with its limited effect on linear electron flow, DMBQ significantly interfered with the reduction of P_700_ in the cyclic pathway, suggesting that it can interact with stroma-soluble electron carriers involved in cyclic electron flow.

Since we aimed at screening different PSII site-directed mutants for their respective ability to divert electrons from PSII to DMBQ, we had to take into account that differences in efficiency of linear electron flow would necessarily arise between strains after mutagenesis. At steady state, the reduction state of P_700_ is equal to *k*_red_/(*k*_oxi_+*k*_red_), where *k*_red_ is the light-induced P_700_ reduction rate and *k*_oxi_ the oxidation rate. Assuming that *k*_oxi_ is fully determined by the light intensity and marginally affected by the addition of quinones (see Supplementary Fig. 1b for the absence of PSI acceptor side limitation above 5 μM of exogenous quinones), *k*_red_ can be computed, as a function of the concentration of quinones and normalized to the reduction rate in the absence of quinones, *k*_red(0)_. Figure 3c,d show the *k*_red_/*k*_red(0)_ ratio computed from the data shown in Fig. 3a,b. In the absence of DCMU, both DCBQ and PPBQ almost completely abolished the electron flux towards P_700_ in the 30–40 μM range, which, by contrast, remained rather insensitive to the addition of DMBQ. In the presence of DCMU (Fig. 3d), we note that, at low-concentration of quinones, DMBQ impacted more efficiently the reduction state of P_700_ than the two other quinones. As expected from its mediocre partitioning within the thylakoid membranes, DMBQ would then readily interact in the stroma with a soluble electron carrier such as, for example, ferredoxin or ferredoxin-NADP^+^ reductase, whereas the action of DCBQ and PPBQ would still be at the level of the intramembrane plastoquinone pool.

### Redesigning PSII to increase reactivity of Q_A_°^−^ with DMBQ

**Figure 4:**
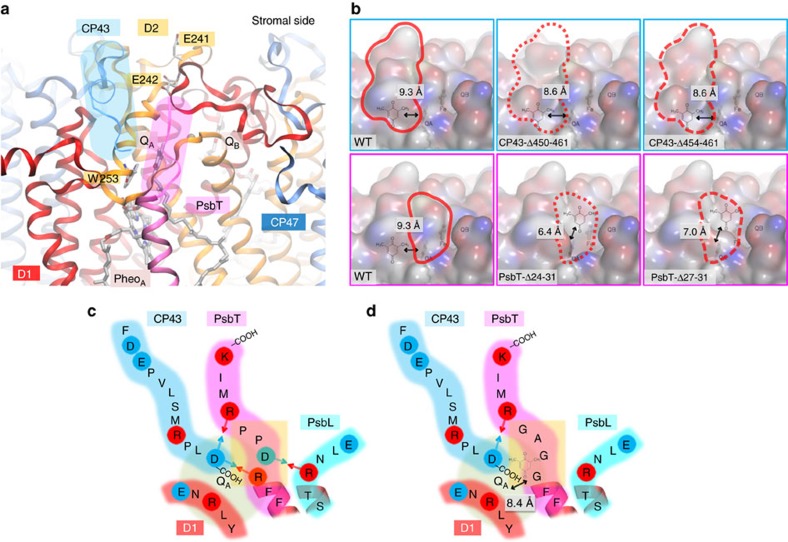
Peptide environment of the Q_A_ binding pocket in *C. reinhardtii* highlighting putative targets for site-directed mutagenesis. The structure of PSII and its mutated variants was based on the PDB entry 3ARC (ref. 26) and built with the SWISS-MODEL workspace^44^,^45^. (**a**) Three dimensional structure of the Q_A_ binding pocket of PSII. The targets for point mutation or truncation in D2 are marked in orange rectangles, and the C-terminal ends of CP43 and PsbT are highlighted in blue and pink rounded rectangles, respectively. (**b**) Surface structure of PSII upon truncation of CP43 or PsbT. Red outlines highlight the truncated region of CP43 (upper panel) or PsbT (lower panel). On each view, the number shows the shortest distance measured between the conjugated ring of Q_A_ and the accessible surface of PSII. It is of note that the I30 and K31 residues of PsbT are not shown as they are absent in the crystal structure. (**c**) Illustration of the 24-RDPP-27 peptide of PsbT and its surroundings. Positively and negatively charged amino acids are circled in red and blue, respectively. Arrows represent putative electrostatic interactions between amino acids of opposite charges. (**d**) Substitution of the 24-RDPP-27 peptide for GGAG results in loss of electrostatic interaction of this part with its neighbouring subunits. The number shows the shortest distance between the conjugated ring of Q_A_ and the water-excluded surface of PSII, determined from the model structures.

To increase the access of stromal exogenous electron acceptors to Q_A_°^−^, we selected several sites of the D2, CP43 and PsbT subunits (encoded by the *psbD*, *psbC* and *psbT* genes, respectively) for mutagenesis, based on the PSII structure modelled after that from *Thermosynechococcus vulcanus*^27^. The environment of the Q_A_ site is well conserved with only one amino acid difference between the two organisms in a 10-Å sphere around Q_A_ (Fig. 4a). We targeted two glutamates of the D2 protein, E241 and E242, exposed to the stromal surface and presumably negatively charged at physiological pH. Another target was the D2-W253 residue, whose mutation may affect the lifetime of Q_A_°^−^ because it is adjacent to Pheo_A_ and Q_A_. Besides these single amino-acid substitutions, we truncated several peptides covering the stromal side of the Q_A_ binding pocket. Increased accessibility was accessed by the shortest distance between the Q_A_ conjugated ring and the water-excluded surface. This distance is decreased by a maximum 2.9 Å in the Δ24–31 truncation of the PsbT subunit, promoting a potential increased rate by a factor of ∼55 (ref. 28). Docking of DMBQ into those newly accessible surfaces corroborates this potential rate acceleration (Supplementary Table 2).

By truncating PsbT, we also aimed at an increased peptide flexibility either by breaking electrostatic interactions between C-terminal sequences of the CP43 and PsbT subunits, or by eliminating two prolines that rigidify the protein backbone. We therefore considered three options: (1) the complete deletion of the C-terminal part from position R24 to K31 (the C-terminus), (2) a shorter deletion from P27 to K31, which preserves the 24-RDP-26 sequence and thus the interactions between PsbT-R24 and CP43-D461 on the one hand, and between PsbT-D25 and PsbL-R15 on the other hand, both of which may stabilize the structure, (3) the substitution of the 24-RDPP-27 tetrapeptide by glycine–glycine–alanine–glycine (GGAG). This would suppress the above-mentioned interactions and may loosen the structure in the vicinity of Q_A_, while preserving the C-terminus sequence of PsbT and, in particular, PsbT-R28 which interacts with CP43-D461 and may thus stabilize this side of the putative entry pathway to Q_A_ (Fig. 4c,d). According to our models in Fig. 4, the most favourable case regarding the accessibility to Q_A_ is the Δ24–31 truncation, followed by the Δ27–31 truncation and the GGAG substitution (Fig. 4b,d). All these mutations, associated with an antibiotic resistance cassette for the selection of transformed cells, were introduced in the chloroplast genome of the WT strain by biolistic transformation. Transformants were all, except that carrying the *psbD*(W253A) mutation, capable of phototrophic growth, although the phototrophic growth capability of the *psbC*(Δ454–461), *psbD*(W253H) and *psbD*(W253Y) mutant strains was significantly reduced (Supplementary Fig. 2).

### Characterization of the mutated strains

**Figure 5:**
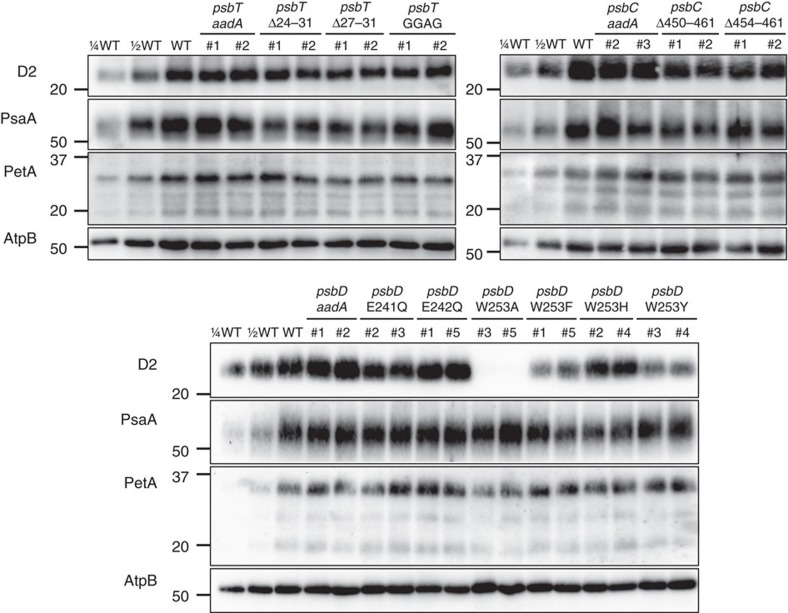
Accumulation of photosynthetic complexes in the mutant strains. Cells were grown in TAP medium at 25 °C under LED white light (8 μmol photons m^−2^ s^−1^) and collected at the mid-log phase. Two independent lines are shown for each construct. Protein samples were loaded on an equal chlorophyll basis (0.5 μg per lane), and a dilution series of WT samples is shown for semi-quantitative comparison. Antibodies against essential subunits of PSII (D2), PSI (PsaA), cytochrome *b*_6_*f* (PetA) and ATP synthase (AtpB) probed the accumulation of the respective photosynthetic complexes. Numbers on the left side of the blots are molecular weights in kD. See Supplementary Fig. 9 for the uncropped blot images.

The relative abundance of PSII in the above mutants was analysed by immunoblotting their content in D2, one of the two major reaction centre II subunits (Fig. 5). None of the *psbT*-C-terminus mutants showed significant changes in their PSII content, but deletion of the CP43 C-terminus resulted in a two-fold decrease in the relative abundance of PSII. Surprisingly, the PSI content was somehow affected in these strains. Polar effects due to *aadA* insertion downstream of the *psbC* gene upon gene transformation might be responsible for these changes. In the *psbD* mutant strains, the E241Q, E242Q and W253H substitutions hardly affected the PSII content whereas it was drastically diminished in the W253Y and W253F mutants and remained below detection in strain W253A. We noted some variation in the accumulation of the *b*_6_*f* complex, probed via its PetA product that did not correlate with the changes in abundance of PSII.

We also measured the PSII/(PSI+PSII) ratio using the light-induced absorbance change at 520 nm, which is proportional to the number of light-induced charge separation^29^,^30^. Overall, the results were consistent with the immunoblot analysis, with the exception of the *psbD*(W253H) mutant that showed only 50–60% PSII activity despite a wild-type content in PSII proteins (Table 1).

We then characterized the various strains using chlorophyll fluorescence analysis (Table 1). The *F*_V_/*F*_M_ value, which is a measure of the maximum quantum efficiency of PSII, expectedly decreased according to the decrease in PSII/(PSI+PSII) ratio. The (*F*_M_−*F*_S_)/*F*_V_ value, which equals the PSII operating efficiency over the PSII maximal efficiency^31^, did not decrease as much as the *F*_V_/*F*_M_ value, thus indicating that the decrease of the PSII to PSI ratio to about 50% in the case of the *psbC* mutant strains did not affect the overall light-induced flux, at the light intensity used for this experiment (26 μmol photons m^−2^ s^−1^).

The mutations introduced in the Q_A_ binding pocket and its surroundings may also affect the midpoint potential of Q_A_/Q_A_°^−^. Changes in this parameter may be assessed by measuring the lifetime of Q_A_°^−^ in the presence of DCMU, although it does not provide an absolute measure of the midpoint potential^32^. Indeed, Q_A_°^−^ lifetime depending on the free energy gap between the P_680_°^+^Q_A_°^−^ and P_680_°^+^Pheo°^−^ radical pairs should be affected by variations in the midpoint potential of the Q_A_/Q_A_°^−^ couple, assuming that the midpoint potentials of the other redox couples involved are not affected in a compensatory manner^32^,^33^. We thus measured the lifetime of Q_A_°^−^ after a single-turnover light flash in the presence of DCMU. The intensity of the laser flash was kept low enough to induce ≤15% of the maximum fluorescence changes. Under such conditions, fluorescence changes are linearly correlated to changes in the fraction of Q_A_°^−^ (refs 34, 35), whose lifetime can be reliably assessed from the decay of fluorescence yield. Table 1 shows that Q_A_°^−^ lifetime was similar in the Δ*psbT* strain and in the WT and only marginally affected in the three *psbT*-C-terminus mutants. C-terminal truncation of the CP43 protein and substitution of E242 by glutamine in the D2 protein somewhat slowed down the re-oxidation of Q_A_°^−^. The D2-W253 site turned out to be an important determinant of the midpoint potential of Q_A_/Q_A_°^−^, as its mutation severely affected the lifetime of Q_A_°^−^ from about two times shorter (W253F) to 5 and 30 times longer (W253Y and W253H) than in the control strain *psbD*(*aadA*) (see Methods for details regarding the construction of the control strain). We note here that, owing to the impressive stability of Q_A_°^−^ in the *psbD*(W253H) mutant strain, a significant fraction of Q_A_ may be in the reduced state in the dark. This precludes a reliable assessment of PSII/(PSI+PSII) by the measure of the light-induced absorbance changes at 520 nm, thus explaining the above-described apparent discrepancy between the functional and biochemical determination of the PSII content in that strain.

### Efficiency of electron transfer to DMBQ in mutated strains

**Figure 6:**
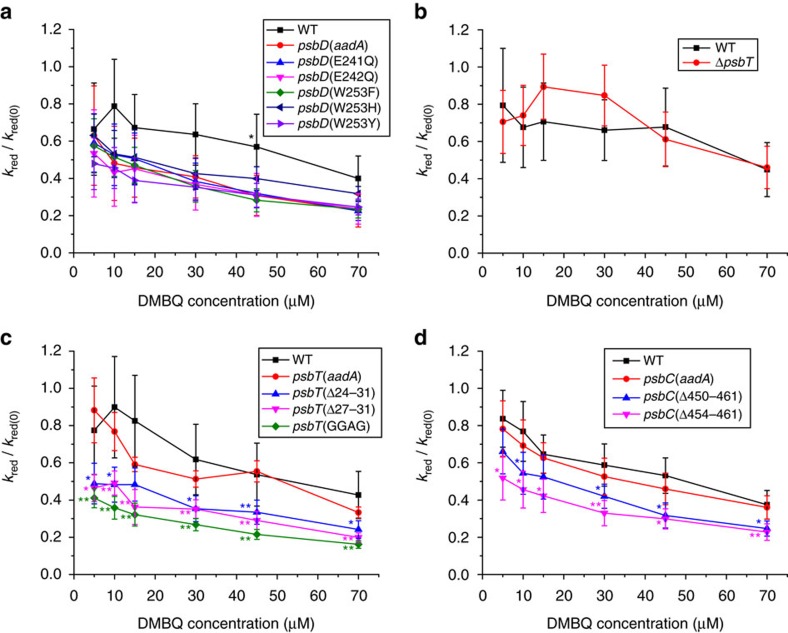
Comparison of the efficiency of electron extraction by DMBQ in the mutant strains. The efficiency of electron extraction was measured in the *psbD* (**a**), *psbT* deletion (**b**), *psbT*-C-terminus (**c**) and *psbC*-C-terminus (**d**) mutant strains by the normalized *k*_red_/*k*_red(0)_ ratio determined from the P_700_ absorbance changes shown in Supplementary Fig. 3. Asterisks indicate a statistically significant difference with respect to the corresponding *aadA* control strain or a significant difference with respect to WT for the Δ*psbT* strain using a two-tailed *t*-test. *: 0.001<*P*<0.05, **: *P*<0.001. Error bars are s.d. of at least three independently performed experiments.

As discussed above, we used the normalized *k*_red_/*k*_red(0)_ ratio to estimate the relative efficiency of light-induced electron flux toward DMBQ in the various mutants. The rationale for this approach is saliently illustrated by the contrasting features of Supplementary Fig. 3a and Fig. 6a, which respectively show the raw P_700_ reduction ratio and the *k*_red_/*k*_red(0)_ ratio as a function of [DMBQ]. Albeit the overall lower electron fluxes from PSII to PSI (Supplementary Fig. 3a), the relative efficiency in the D2-W253 mutants was not higher than that in the control strain (Fig. 6a). We also note that the normalized reduction rate of P_700_ was higher in WT than in the *psbD*(*aadA*) and most of the *psbD* mutant strains. This may stem from genetic drift upon several rounds of subcloning of the transformants on spectinomycin-containing growth medium (see ref. 36 for discussion of the wild type concept in Chlamydomonas), as well as from limited changes in chloroplast gene expression, when introducing the *aadA* gene downstream of the *psbD* gene.

Although we observed no significant differences in the *k*_red_/*k*_red(0)_ ratio between the Δ*psbT* and WT strains (Fig. 6b and Supplementary Fig. 3b), the three *psbT*-C-terminus mutant strains displayed a lower normalized reduction rate than the control strain in the presence of DMBQ (Fig. 6c). This suggests that modifying the C-terminal part of PsbT increased the accessibility of Q_A_°^−^ to DMBQ, thereby generating a man-made electron transfer pathway competing with the natural flow between PSII and PSI. In addition, the data obtained in the absence of exogenous quinones (Supplementary Fig. 3c) showed that, in the conditions tested so far, the photosynthetic capacities of these strains are not compromised by the mutations. The *psbC* mutants looked less promising since, despite their higher sensitivity to the addition of quinones in terms of electron flow from PSII to PSI (Fig. 6d), they had a somewhat reduced PSII content (Table 1) that suggests a compromised stability of the protein that may become critical in other experimental conditions. We note that, when assessed in the presence of DCMU, we found no further effect of DMBQ on the electron transport around PSI, as indicated by the *k*_red_/*k*_red(0)_ ratio (Supplementary Fig. 4), which excluded that there were additional electron sources for DMBQ reduction within cyclic electron flow in these mutants. We thus chose to further characterize the various *psbT* mutants since they turned out to be most promising regarding the design of a novel electron transfer from Q_A_°^−^ to exogenous and stromal electron carrier.

### Extraction of electrons from Q_A_°^−^ in the presence of DCMU

**Figure 7:**
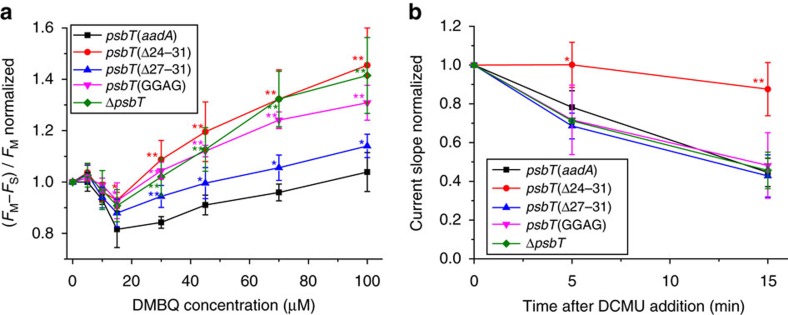
Evidence of electron transfer from Q_A_°^−^ to DMBQ in the *psbT* mutant strains. (**a**) Direct electron transfer from Q_A_°^−^ to DMBQ as revealed by chlorophyll fluorescence analysis under dim light in the *psbT* mutant strains. The ratio of (*F*_M_−*F*_S_)/*F*_M_ normalized to the level in the absence of DMBQ was measured in the presence of 10 μM DCMU at actinic light of 0.6 μmol photons m^−2^ s^−1^. The larger normalized (*F*_M_−*F*_S_)/*F*_M_ ratio in these mutant strains relative to the *aadA* control strain suggests that additional electron transfer from Q_A_°^−^ to DMBQ competes with fluorescence. Asterisks indicate a statistically significant difference from the level of the *aadA* control strain using a two-tailed *t*-test. *: 0.001<*P*<0.05, **: *P*<0.001 (mean±s.d.; *n*=6). (**b**) Chronoamperometic measurements to estimate the sustained electron transfer from Q_A_°^−^ to DMBQ in the presence of DCMU. The slope of current rise, which relates to the reduction rate of DMBQ, was estimated at 5 and 15 min after the addition of DCMU and normalized to the slope at 2.5 min before the DCMU addition (see Methods for details). Asterisks indicate a statistically significant difference from the level of the *aadA* control strain using a two-tailed *t*-test. *: 0.001<*P*<0.05, **: *P*<0.001. Error bars are s.d. of at least four independently performed experiments.

To further determine whether DMBQ genuinely harvests electrons from Q_A_°^−^ by approaching the stromal side of the Q_A_ binding pocket in the *psbT* mutants, we designed the following experiment. Algae, first incubated in darkness with DCMU to inhibit electron transfer between Q_A_ and Q_B_, were subjected to dim light (0.6 μmol photons m^−2^ s^−1^) to generate a steady-state situation in which Q_A_°^−^ should accumulate only in a fraction of PSII. This steady-state concentration of Q_A_°^−^ in the presence of DCMU results from the balance between its rate of light-induced formation and its rate of charge recombination with the oxidizing equivalent stored in the oxygen evolving complex. Provided the light intensity, which determines the rate of Q_A_°^−^ formation, is kept commensurate with the charge recombination rate, Q_A_°^−^ will accumulate only in a fraction of PSII and the steady-state fluorescence level, *F*_S_, will be lower than *F*_M_. Under such conditions, the exogenous electron acceptor capable to oxidize Q_A_°^−^ via our engineered pathways should decrease the steady-state concentration of Q_A_°^−^ and, from an experimental stand-point, *F*_S_. Figure 7a shows the dependence on (DMBQ) of the (*F*_M_−*F*_S_)/*F*_M_ ratio normalized to its value in the absence of quinones. Besides a transient decrease at low concentration of DMBQ, the increase in (*F*_M_−*F*_S_)/*F*_M_ with increasing (DMBQ) was clearly more pronounced in the Δ*psbT* and *psbT*(Δ24–31) than in the control strain, reaching values close to 1.4 at the maximum concentration tested. The *psbT*(GGAG) mutant also displayed a significant increase in (*F*_M_−*F*_S_)/*F*_M_ ratio up to about 1.2, as did the *psbT*(Δ27–31) strain, albeit to a much lesser extent.

### Chronoamperometric measurements

**Figure 8:**
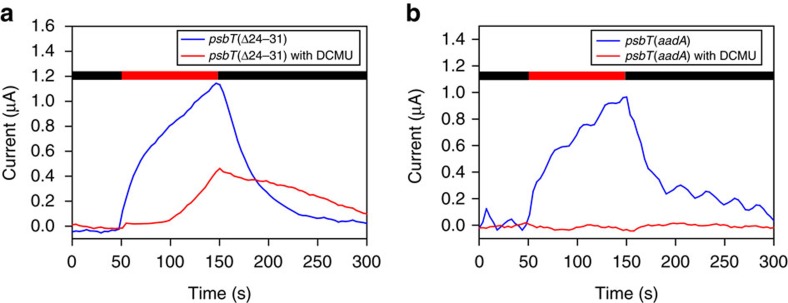
Pre-incubation with DCMU supports a direct electron transfer from Q_A_°^−^ in the *psbT*(Δ24–31) mutant strain. (**a**) A pre-incubation with DCMU (10 μmol l^−1^) in the *psbT*(Δ24–31) mutant strain does not fully inhibit the production of a photo-induced current (illumination is indicated by the red horizontal bar) due to the electron transfer between PSII and DMBQ. (**b**) The current recorded in the control strain is fully inhibited by a pre-incubation with DCMU.

We then used chronoamperometry (see Methods for details) to assess the ability of DMBQ to divert electrochemical current from the light-induced electron transfer. Representative curves are displayed in Supplementary Fig. 5. We determined the reaction of the light-induced current to the addition of DCMU, the rationale being that only those mutants allowing access of exogenous DMBQ to Q_A_ will generate a light-induced current, when electron transfer from Q_A_ to Q_B_ was inhibited. Figure 7b shows the variations of the slope of the amperometric signal versus time after addition of DCMU. To take into account the differences between strains, the slopes were normalized to their values before DCMU addition. In the control strain *psbT*(*aadA*), the normalized slope of the amperometric signal expectedly declined after addition of DCMU. We note that this decline developed in the range of 10–20 min, which reflects the time necessary for the electrode to fully electrolyse the sample volume of the set-up (see Methods and Discussion). The reaction of the amperometric signal of the *psbT* mutants to the addition of DCMU was similar to that of the *psbT*(*aadA*) control, with the remarkable exception of the *psbT*(Δ24–31) strain: the light-induced exogenous current proved remarkably resilient to the addition of DCMU. We then performed another type of experiment in which the strains were incubated with DCMU well before the onset of illumination (Fig. 8). In this case, the set-up was adapted to analyse the current production at more short times (a few minutes instead of a few hours). Here again the *psbT*(Δ24–31) strain remained capable of producing a photocurrent in presence of DCMU albeit of lower intensity then in the absence of the inhibitor, while the photocurrent in the control strain was fully suppressed by a preincubation with DCMU. Importantly, the *psbT*(Δ24–31) mutant was also short-listed after the two previous screens we implemented, either when assessing the electron transfer from Q_A_°^−^ to DMBQ in presence of DCMU or electrons diverted to DMBQ upstream of P_700_. Thus the chronoamperometric measurements provided further support to the conclusion that Δ24–31 truncation of the PsbT subunit allows electron flow to DMBQ that by-passes the natural Q_A_°^−^ to Q_B_ pathway.

## Discussion

We aimed at establishing a proof-of-principle device that would use intact engineered unicellular algae—in the present case *Chlamydomonas reinhardtii*—to produce a photocurrent without compromising their ability to perform photosynthesis. Producing a photocurrent out of a living cell may of course be the result of many processes, which may be difficult to properly identify, in such a complex multimolecular and multicompartment context. In this respect, the 12 PSII mutant strains in this study provided very contrasting results, once screened for a preserved intrinsic photosynthetic activity and for their ability to transfer electron to DMBQ that would sustain a usable photocurrent. These successive screens lead us to retain one mutant that satisfied the two criteria above therefore standing as the corner stone of future development and improvement of photovoltaic devices along the above described strategy. This study also raises a number of issues worth further investigation, as discussed below.

Several studies have suggested that methyl-substituted benzoquinones, compared with chloro-substituted benzoquinones, have generally less efficient support for light-induced oxygen evolution^17^,^18^. The present fluorescence analysis shows that DMBQ is a far less efficient PSII electron acceptor than DCBQ. Its lower reactivity with the plastoquinone pool may be partly due to its relatively negative midpoint potential compared with that of the two other quinones we studied^25^, but it is also consistent with a lower partitioning in the thylakoid membranes as suggested by the remarkably low quenching capacity of DMBQ (Fig. 2b and ref. 25). In contrast, DMBQ proved as efficient an electron acceptor as DCBQ and PPBQ when interfering with cyclic electron flow, as measured by the P_700_ re-reduction in the presence of DCMU (Fig. 3d), indicating that it reaches the stromal compartment where it may act as an electron acceptor. Therefore, we selected DMBQ as the proper redox mediator to implement our strategy.

The next step was to design a mutant in which exogenous DMBQ would approach Q_A_°^−^ from the stroma and divert a fraction of the photo-induced electron flow, without impacting the photosynthetic activity in its absence. Substitution of D1-K238 by glutamate in *Synechocystis* was shown to promote the reduction of cytochrome *c* by Q_A_°^−8^. Thylakoids of this mutant strain generated a greater photo-induced current with respect to WT in the presence of exogenous cytochrome *c*, but its addition as a redox mediator between PSII and the electrode surprisingly turned out to be dispensable^7^,^8^. Here, our engineering specifications excluded any purification step thereby imposing the use of an amphiphilic redox mediator able to cross the cell membranes. Thus, the parameters that we had to tune were either the lifetime of Q_A_°^−^ or the accessibility of Q_A_°^−^ to a stromal and amphiphilic electron carrier, which is represented by the distance between the two since distance largely determines electron tunnelling rates^28^,^37^.

Owing to the short distance between the tryptophan in position 253 of the D2 protein and the Q_A_ site, we had anticipated that its substitution may affect the semiquinone lifetime. Indeed, we observed a surprisingly long-lived semiquinone radical at the Q_A_ site when tryptophan was changed to histidine or tyrosine but not to phenylalanine. This suggests that changing a non-polar aromatic residue to a polar one at position 253 considerably stabilizes the semiquinone radical, an effect that would indicate a significant shift in the midpoint potential of the Q_A_/Q_A_°^−^ to more positive values^38^. We had hypothesized two possible contributions to a better extraction of electrons at the Q_A_ site: (1) a longer-lived Q_A_°^−^ species and (2) a shorter electron transfer distance. Hypothesis (1) found no further support since the *psbD*(W253H) mutant did not sustain a higher extraction efficiency of electrons by DMBQ despite its very long-lived Q_A_°^−^ species, thirty times longer than in the control strain (Table 1 and Fig. 6). By contrast, the promising results with *psbT*-C-terminus mutants suggest that the main contribution to a better electron extraction at the Q_A_ site would rather come from an increased access to exogenous quinones. In other words, peptides covering the Q_A_ molecule on the stroma surface prevent redox mediators from harvesting electrons on the stromal side unless they are truncated or modified. We targeted the PsbT subunit because previous studies of the Δ*psbT* strain of *C. reinhardtii* had shown it was still able to grow phototrophically, although cell growth was severely impaired in high-light conditions^39^,^40^. The present *psbT*-C-terminus mutant strains also grew phototrophically, and had a similar amount and activity of PSII as the wild type. Accordingly the longest truncation we used—8 amino acids at the C-terminus of PsbT protein (Δ24–31)—did not compromised PSII activity, in stark contrast with the truncation of 8 amino acids at the C-terminus of CP43 protein (Δ454–461) that significantly compromised PSII accumulation.

When using the available X-ray structural data of PSII from *T. vulcanus*^27^, amended for a few amino acid substitutions present in *C. reinhardtii*, we obtained distances between Q_A_ and the modified molecular surface of the various mutants that correlated nicely with electron withdrawal by DMBQ. Docking simulation yielded several docking sites based on the estimation of binding free energy that corroborate the reduction of accessible distances, suggesting that the new formed crevices may be accessible and used by quinones. Nevertheless, the Δ24–31 truncation of the PsbT subunit was the most promising candidate using either of the two approaches, and, from all PSII mutants we examined, the *psbT*(Δ24–31) mutant showed the most efficient extraction of electrons as proved by (1) the highest re-reduction efficiency of P_700_ in the absence of DCMU, (2) the highest oxidation efficiency of Q_A_°^−^, and (3) the highest electrochemical current that could be generated in the presence of DCMU.

The screen for high-extraction efficiency of electrons and for strong interaction with Q_A_°^−^ were respectively conducted by measuring the redox absorbance change of P_700_ in the absence of photosynthesis inhibitor and chlorophyll fluorescence in the presence of DCMU. While the latter experiment assays the accessibility of Q_A_°^−^ to DMBQ, the former assays the ability of DMBQ to compete with Q_B_ in the oxidation of Q_A_°^−^. Thus, even though a mutation may provide an improved accessibility to the Q_A_ site, the electron donation rate to DMBQ may remain too low to compete with the transfer to Q_B_. That was clearly observed when comparing the *psbT* mutants. The assay conducted in the presence of DCMU would appoint the Δ*psbT*, *psbT*(Δ24–31) and *psbT*(GGAG), as the three mutants providing the best access to exogenous DMBQ. According to the P_700_ data, however, only the latter two displayed significant diversion of electrons from P_700_ reduction in the presence of DMBQ. Since the PsbT subunit has a transmembrane orientation in the vicinity of a cluster of carotenoids at the PSII dimer interface, its absence may impact neighbouring protein subunits and cofactors and result in a lower stability of PSII lacking PsbT in adverse conditions, as was shown earlier by its increased photosensitivity^39^,^40^. Further *in vitro* experiments using the engineered PSII reaction centres harbouring modified PsbT sequences should allow one to fully characterize their redox and kinetic properties, when assessed in the presence of DMBQ.

For our chronoamperometric measurements, we used an adapted set-up to extract photosynthetic electrons from an algae population by means of exogenous DMBQ acting as an electron carrier to a large carbon electrode surface. The resulting photocurrent under illumination was in the range of 10–20 μA. Comparison of these results with those from studies using non-intact or immobilized cells on other types of electrodes should be taken with care. Still the performance is often in the same range. As an example, the current density reported by Sekar *et al*.^12^ using immobilized cyanobacteria is close to 2,000 mA m^−2^ with 2 mM quinones. This is of the same order of magnitude as the present work (10–100 mA m^−2^ with 30 μM quinones), given that changes in configuration (adsorbed organisms, specific surfaces and so on) prevent a more accurate comparison.

The specific resiliency of the DMBQ-mediated photocurrent upon addition of DCMU to the *psbT*(Δ24–31) mutant demonstrates the significance of the chronoamperometric measurement regarding extraction of electrons from the Q_A_ site. Such a resiliency was also observed when considering a preincubation with DCMU. However, we noted that our set-up delivered photocurrents in the same range of amplitudes regardless of the quinone added (see Supplementary Fig. 8 for examples) despite their different extraction sites of photosynthetic electrons. These observations suggest a current limited by the mass transfer (convection and diffusion) of DMBQ between the bulk solution and the anode, which leads to many energy losses. Still, our experimental values compare well with an estimation of the photocurrent magnitude resulting from a convection-diffusion limitation (see Methods), which gives values of a few tens μA depending on the thickness of the diffusion layer.

## Methods

### Strains and growth conditions

*C. reinhardtii* strain T222^+^ (hereafter referred as WT), derived from multiple backcrosses of the 137c strain^41^, and the Δ*petA* strain lacking the cytochrome *b*_6_*f* complex^42^ were used in this study. The *psbT* deletion (Δ*psbT*) strain^39^ is a kind gift from Yuichiro Takahashi (Okayama University, Japan). Cells were grown to mid-log phase (2–5 × 10^6^ cells per ml) in Tris-acetate-phosphate (TAP) liquid medium^43^ at 25 °C under white LED light (8 μmol photons m^−2^ s^−1^) before further measurements.

### Molecular modelling and visualization

Homology models of the *C. reinhardtii* PSII and of its mutated variants were based on the PSII crystal structure of *T. vulcanus* (PDB code: 3ARC)^27^ and built with the SWISS-MODEL workspace^44^,^45^. Docking simulation of DMBQ onto the stromal side of the PSII was performed with the AutoDockTools and Autodock Vina programs^46^,^47^. Accessibility distances were calculated with UCSF Chimera^48^. The protein structures were visualized with the VMD program^49^.

### Site-directed mutagenesis of chloroplast-encoded genes

To construct chloroplast transformation vectors for site-directed mutagenesis of the CP43, PsbT, or D2 proteins in *C. reinhardtii*, plasmids encompassing the *psbC*, *psbT* or *psbD* genes were respectively obtained from the Chlamydomonas Resource Center (P-578: *psbC* and P-72: *psbT*) or the pH3P plasmid for *psbD* (ref. 50). To avoid any polar effects of the insertion of the antibiotic resistance cassette used for the selection of transformants, which could hamper the expression of the downstream located genes, we used an engineered *aadA* cassette. We cloned an extra copy of the *psaA* promoter and 5′UTR regions downstream of the strong processing site within the *rbcL* 3′UTR. This was done by megaprime PCR^51^ using plasmid paAKR^52^ as a template and oligonucleotides rbcLCod and psaARev as external primers (see Supplementary Table 1 for the list of the oligonucleotides used in this work), and rbcLRev and psaACod as internal primers. The 494 bp long resulting PCR product, comprising a shortened version of the 3′*rbcL* followed by the *psaA* promoter and 5′UTR regions, was digested with *Pst*I and *Sph*I and cloned into the paAKR plasmid digested with the same enzymes to yield plasmid paAKRaA.

For *psbD* site directed mutagenesis, the pH3P plasmid^50^ was first shortened by digestion with *Pst*I and *Av*rII and religated on itself to create plasmid pH3P_Sh_. The modified *aAKRaA* cassette, excised from plasmid paAKRaA with restriction enzymes *Sma*I and *Eco*RV was introduced into the *Ale*I site of the pH3P_Sh_ plasmid, downstream of and in the same orientation as *psbD* to yield plasmid pH3aAKRaA, used to generate the *psbD*(*aadA*) control strain. Site-directed mutations were constructed by megaprime PCR using plasmid pH3P_Sh_ as a template, the external primers psbD-mut-EF/ aadA-ER and the mutagenic oligonucleotides psbD-XX-IF/psbD-XX-IR (where XX corresponds to the mutations listed in Supplementary Table 1b). The final PCR product (1,562 bp) was digested by *Bst*EII and *Aar*I and cloned into the pH3aAKRaA vector digested by the same enzymes to yield transformation vectors pH3aAKRaA(XX).

For *psbT* site directed mutagenesis, the P-72 plasmid contains a 4.4 kb *Eco*RI fragment of the *C. reinhardtii* chloroplast genome encompassing the *psbT* coding sequence cloned in pUC8 and was obtained from the Chlamydomonas Resource Center. *Cla*I and *Age*I restriction sites were introduced downstream of the *psbT* 3′UTR by megaprime PCR using the external primers psbT-aadA-EF/psbT-aadA-ER and the mutagenic primers psbT-aadA-IF/psbT-aadA-IR. The resulting 1,228 bp amplicon was digested with *Ban*II and *Pst*I and cloned into the P-72 vector digested with the same enzymes to generate P-72_AC. The *aAKRaA* resistance cassette, excised from plasmid paAKRaA by digestion with *Cla*I and *Ava*I, was cloned into the *Cla*I- *Age*I digested plasmid P-72_AC to yield the P-72aAKRaA plasmid, used to generate the *psbT*(*aadA*) control strain. Mutations in the C-terminal end of PsbT were introduced by megaprime PCR using the external primers psbT-aadA-EF and aadA-ER and the mutagenic primers psbT-YY-IF and psbT-YY-IR, where YY designates the mutations listed in Supplementary Table 1c. The resulting 1,047 bp amplicon, digested with *Ban*II and *Bst*EII, was cloned into the P-72aAKRaA plasmid digested with the same enzymes to generate the P-72aAKRaA(YY) chloroplast transformation vectors.

For *psbC* site directed mutagenesis, the P-578 plasmid, obtained from the Chlamydomonas Resource Center, harbours a 10 kb *Eco*RI fragment of the chloroplast genome encompassing the last 173 codons of *psbC*, cloned in pBR328. This plasmid was first shortened by digestion with *Sna*BI and *Tth*111I and religated on itself to yield plasmid P-578_Sh_. *Mfe*I and *Xma*I restriction sites were then introduced downstream of the *psbC* 3′UTR by megaprime PCR using psbC-aadA-EF/psbC-aadA-ER as external primers, and the mutagenic oligonucleotides psbC-aadA-IF/psbC-aadA-IR (see Supplementary Table 1d). The resulting 1,448 bp amplicon was digested with *Pml*I and *Xcm*I and cloned into the P-578_Sh_ vector digested by the same enzymes to generate plasmid P-578_Sh__MX. The *aAKRaA* resistance cassette, excised from plasmid paAKRaA by *Eco*RI and *Xma*I, was cloned between the *Mfe*I and *Xma*I sites of P-578Sh_MX to yield the P-578aAKRaA plasmid, used to recover the *psbC*(*aadA*) control strain. Truncations of the PsbC C-terminal ends were generated by PCR using the psbC-stop-R oligonucleotide together with primers psbC-F450stop-F or psbC-V454stop-F. The resulting 794 bp PCR products were digested by *Pml*I and *Blp*I and cloned into the P-578aAKRaA plasmid digested by the same enzymes to create the psbC(F450st) and psbC(V454st) transformation vectors.

All constructs were sequenced before transformation into the *Chlamydomonas* chloroplast genome to assess the presence of the desired mutations in an otherwise wild-type sequence. Mutations were introduced in WT *C. reinhardtii* cells by biolistic transformation^53^ using a home-built helium-driven biolistic gun^54^. The *aadA* strains containing the 5′*psaA*-*aadA-3′rbcL* gene without site-specific mutations were used as a control which shares with the mutant strains the same *aadA* insertion. Transformants were selected on spectinomycin-containing TAP solid medium plates (100 μg ml^−1^) and further sub-cloned until they reached homoplasmy on TAP solid medium supplemented with 500 μg ml^−1^ spectinomycin. Homoplasmy was checked by RFLP analysis of specific PCR products. At least three independent transformants were analysed for each constructs; variations between independent clones proved negligible.

### Immunoblot analysis

Cells grown in TAP liquid medium were centrifuged at 4,000*g* for 5 min at 20 °C and resuspended in 0.1 M DTT, 0.1 M Na_2_CO_3_, 15% (w/v) glycerol, 1 × proteinase inhibitor cocktails (Roche) and frozen in liquid nitrogen. After addition of SDS (2% final), samples were heated at 95 °C for 1 min. The insoluble material was removed by centrifugation at 12,800*g* for 20 min, and proteins were loaded on an equal chlorophyll basis on a Mini-PROTEAN electrophoresis system (Bio-Rad) and separated by SDS–polyacrylamide gel electrophoresis (SDS–PAGE). For immunoblot analysis, proteins were transferred onto nitrocellulose membranes, and the membranes were blocked with 5% (w/v) skim milk in a phosphate-buffered saline (PBS) solution plus 0.1% (v/v) Tween 20 (Sigma-Aldrich). The target proteins were probed with primary antibodies and then incubated with horseradish peroxidase-conjugated anti-rabbit IgG antibodies (Catalogue number: W4011, Promega) at a dilution ratio of 1:100,000. The primary antibodies against the PSII subunit D2 (Catalogue number: AS06 146; used at a dilution ratio of 1:50,000) and the PSI subunit PsaA (Catalogue number: AS06 172; used at a dilution ratio of 1:10,000) were purchased from Agrisera. Antibodies against cytochrome *f* (PetA; used at a dilution ratio of 1:5,000) were prepared using purified cytochrome *f* injected to rabbits^54^, and those against the ATP synthase subunit β (AtpB; used at a dilution ratio of 1:5,000) were kindly provided by C. Lemaire (Centre de Génétique Moléculaire, Gif-sur-Yvette, France)^55^. Immuno-reactive proteins were detected with an Amersham ECL Select detection reagent (GE Healthcare) and visualized using the ChemiDoc XRS+ System (Bio-Rad). The uncropped blot images of Fig. 5 are displayed in Supplementary Fig. 9.

### Chlorophyll fluorescence and spectroscopic analyses

Cells grown in TAP liquid medium were centrifuged at 4,000*g* for 5 min at 20 °C and resuspended in 20 mM HEPES pH 7.2, 10% (w/v) Ficoll PM400 (Sigma-Aldrich). Cells were then vigorously shaken at 300 r.p.m. for 30 min in the dark before spectroscopic measurements. Light-induced changes of absorbance and of chlorophyll fluorescence were measured with a JTS-10 spectrophotometer (Bio-Logic). Detection and actinic lights were provided by LEDs, and a short (5 ns) saturating light flash was provided by a dye laser (640 nm) pumped by a second harmonic Nd-YAG laser (Minilite, Continuum). Interferential filters were used to transmit detection light at selected wavelengths. The 6-mm bandpass glass filters (BG-39, Schott) were placed in front of photodiodes to cut off most of the excitation light.

The ratio of PSII to (PSI+PSII) was computed from absorbance changes measured at 520 nm (10 nm width at half maximum) 100 μs after an actinic flash: we subtracted signals obtained after adding 10 μM DCMU plus 1 mM hydroxylamine to pre-illuminated algae, yielding only one electrogenic charge transfer event per PSI, from those obtained with untreated algae that correspond to one electrogenic charge transfer event per PSI+PSII^29^,^30^. The P_700_ redox levels were calculated from absorbance changes measured at 705 nm (6 nm width at half maximum) corrected by subtraction of absorbance changes measured at 730 nm; the P_700_ reduction ratio is 1 minus the light-induced oxidation ratio of P_700_ (see Supplementary Fig. 1a for the calculation of the oxidation ratio of P_700_). The latter ratio is defined as the light-induced oxidation level of P_700_ divided by the maximal amplitude of P_700_ oxidation, which corresponds to the complete oxidation of P_700_ after a 10-ms excitation light pulse minus its relaxation level in the dark^56^,^57^.

### Electrochemical analysis

The chronoamperometric measurements were performed in an adapted three-compartment cell using a QuadStat potentiostat supplemented with an e-corder data recorder (eDAQ). Vitreous carbon foam (ref. 398–733–69, Goodfellow Cambridge, UK) was used as a working electrode (∼0.25 cm^3^) and soaked in PBS solution (ref. 18912, Life Technologies) overnight before measurements. A platinum wire and an Ag/AgCl wire were used as counter and reference electrodes, respectively, and both separated with fritted glass of medium porosity. All the measurements were carried out in PBS solution at 25 °C. Control experiments demonstrated that negligible current was generated upon illumination in our set-up when *C. reinhardtii* cells or quinones were not supplemented (Supplementary Fig. 6). The generated current would reach to a steady-state level after ∼1 h of illumination (Supplementary Fig. 7).

For kinetic study of DMBQ reduction, *C. reinhardtii* cells grown in TAP liquid medium were centrifuged at 4,000*g* for 5 min at 20 °C and resuspended in the electrolytic cell containing 8 ml of PBS solution with the chlorophyll concentration adjusted to 4 μg ml^−1^. The solution was stirred with a magnetic stirrer at 350 r.p.m. The biased potential was set at 0.65 V versus Ag/AgCl, and the current was recorded every 2 s. The solution was supplemented with 30 μM DMBQ 5 min before its subjection to the illumination of green LED light (peak emission at 530 nm, 50 nm width at half maximum, 30 μmol photons m^−2^ s^−1^). The rise of current reached a steady-state level after 5–10 min of illumination, and 10 μM DCMU was added 15 min after illumination. The slope of current rise was estimated using the least squares approach to fit the current data in the range of a certain time point±1.5 min to a linear model. Because of the different locations of the electrode and the illumination zone (due the spectro-electrochemical cell geometry), the photocurrent magnitude is expected to be limited by mass-transport-controlled electrochemical re-oxidation (nFACD/δ). Given *n*=2, *F*=96,500 C mol^−1^, *D*=10^−6^ cm^2^ s^−1^ and A=specific electrode surface area given by the supplier=20 cm^2^, typical current values for *C*=30 μmol l^−1^ may range from 10 to 90 μA depending on *δ*=diffusion layer estimated as being in the range of 12.5 (from Levich's law) to 100 μm. The recorded photocurrent values (10–20 μA) are of the same order of magnitude knowing that the maximum current value (90 μA) is an overestimation. In particular, the lowest diffusion layer thickness is probably underestimated by the Levich's approximation, whereas the active quinone concentration is overestimated. Indeed it depends on the actual concentration of oxidized quinones in solution and on the other hand, a significant portion should be sequestered in cellular compartments other than the thylakoid membranes^25^.

Chronoamperometric measurements involving a preincubation with DCMU were performed in an adapted electrochemical cell using a Princeton potentiostat Parstat 2273. An ITO/Au modified glass slide was used as the working electrode. This electrode was obtained by sputtering a thin indium tin oxide (ITO) film (thickness 10 nm) followed by another film of gold (20 nm) onto the glass slide using a sputtering system EMITECH K675X. A PDMS (polydimethyloxane RTV-615; Momentive Performance Materials) piece with a 9 mm diameter hole inside was attached on the ITO/Au modified glass slide by the mean of a treatment with oxygen plasma at 400 mtorr (100 W) for 3 min (Harrick Plasma, NY, USA). The final device thus corresponds to a PDMS well (volume ∼0.5 ml) possessing the ITO/Au working electrode in the bottom. A platinum wire and a Ag/AgCl wire were finally used as the counter and as the reference electrodes, respectively.

C. *reinhardtii* cells were grown in TAP liquid medium and resuspended in minimum medium (chlorophyll concentration adjusted to 4 μg ml^−1^). Solutions were incubated with DCMU (10 μM) and then DMBQ (30 μmol l^−1^; 20 min) or only with DMBQ (30 μmol l^−1^; 20 min) before the experiments were carried out. The working electrode potential was subsequently set at 0.38 V versus Ag/AgCl and the current was recorded every 2.7 s. The actinic light was triggered once a stable baseline is observed. The actinic white light was provided by a Scott KL1500 LCD halogen lamp (50 μmol photons m^−^^2^ s^−1^ spanning the 400–700 nm range). The lamp was switched on 30 min before shutter opening to allow temperature stabilization.

### Data availability

All the data generated during the study are available from the authors.

## Additional information

**How to cite this article:** Fu, H.-Y. *et al*. Redesigning the Q_A_ binding site of Photosystem II allows reduction of exogenous quinones. *Nat. Commun.* **8,** 15274 doi: 10.1038/ncomms15274 (2017).

**Publisher's note:** Springer Nature remains neutral with regard to jurisdictional claims in published maps and institutional affiliations.

## Supplementary Material

Supplementary Information Supplementary Figures and Supplementary Tables

## Figures and Tables

**Table 1:**
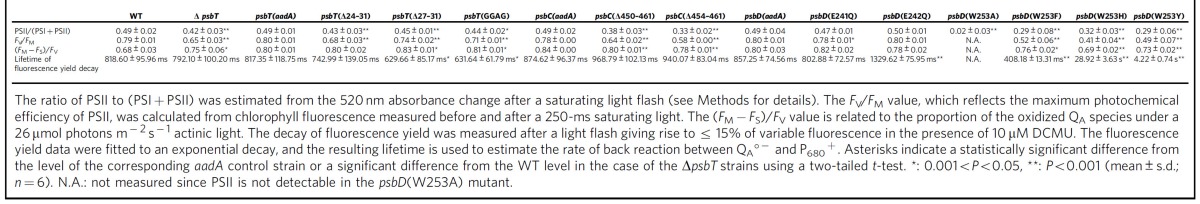
Photosynthesis related parameters of the mutant strains.
