# Supplementary material for: Redesigning the QA binding site of Photosystem II allows reduction of exogenous quinones
Source: Nat Commun. 2017 May 3;8:15274. doi: 10.1038/ncomms15274 (PMC5418674; doi:10.1038/ncomms15274)
Supplement: Supplementary Information — Supplementary Figures and Supplementary Tables [file ncomms15274-s1.pdf]

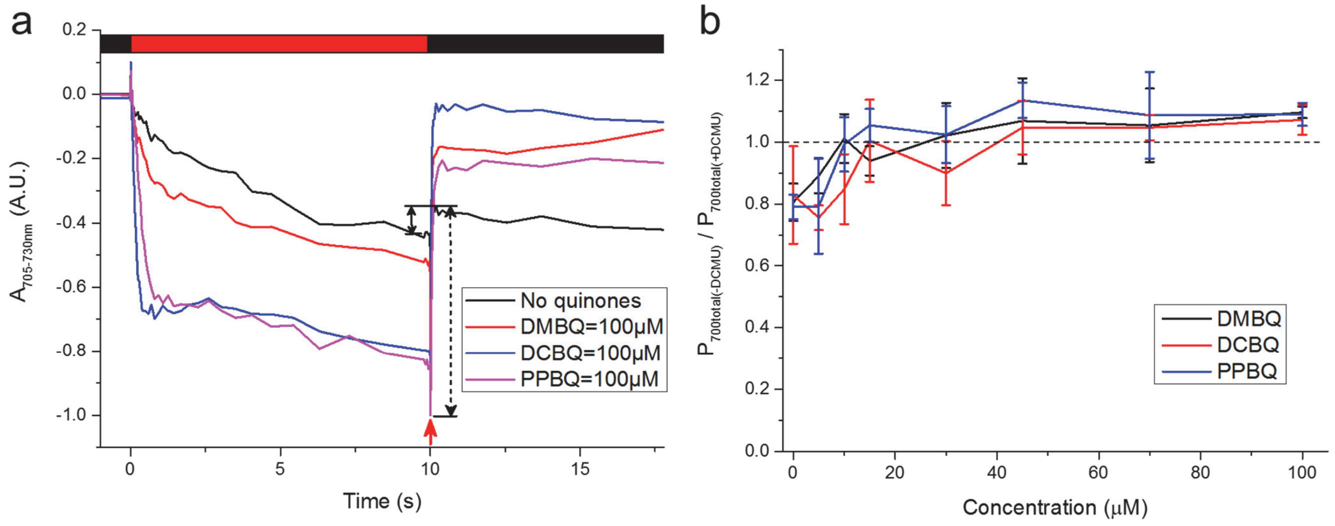

Supplementary Figure 1 | Analysis of light-response P_{700} absorbance changes

(**a**) Representative traces of light-response P_{700} absorbance changes upon the treatment with exogenous quinones. The absorbance changes measured at 705 nm subtracted by the absorbance changes at 730 nm ($A_{705\text{-}730nm}$) are normalized to the lowest value after a saturating light pulse (red arrow). After illumination with actinic light (26 μmol photons m^{-2} s^{-1}) for ~10 s, as indicated by the red horizontal bar, the $A_{705\text{-}730nm}$ absorbance change due to the oxidation of P_{700} reached a steady-state level. After a saturating light pulse, the absorbance change reflects the full population of photo-oxidizable P_{700} and then recovers to a fully reduced state. The oxidation ratio of P_{700} was calculated as the light-induced oxidation level of P_{700} (indicated by the black solid arrow) divided by the maximum amplitude of P_{700} oxidation (indicated by the black dashed arrow). (**b**) Ratio of photo-oxidizable P_{700} ($P_{700total}$) in the absence of DCMU to that in its presence (10 μM). The $P_{700total}$ level was calculated as the complete oxidation of P_{700} after a 10-ms excitation light pulse minus its relaxation level in

the dark. In presence of DCMU, the inhibition of PSII-dependent re-reduction of P700 releases acceptor side limitation of PSI, thus allowing a measure of the total population of P700 in the thylakoid membranes. It is of note that the ratio is close to 1 (indicated by the dashed line) upon supplementation with exogenous quinones above 5 µM, which indicates the absence of PSI acceptor side limitation above this quinone concentration. In these conditions, the stromal PSI acceptors are fully oxidized and the amount of P_{700} that can be photo-oxidized during a saturating pulse reaches the total amount of P_{700} available in the thylakoid membranes, as detected in presence of DCMU under continuous illumination. Error bars are s.d. of at least three independently performed experiments.

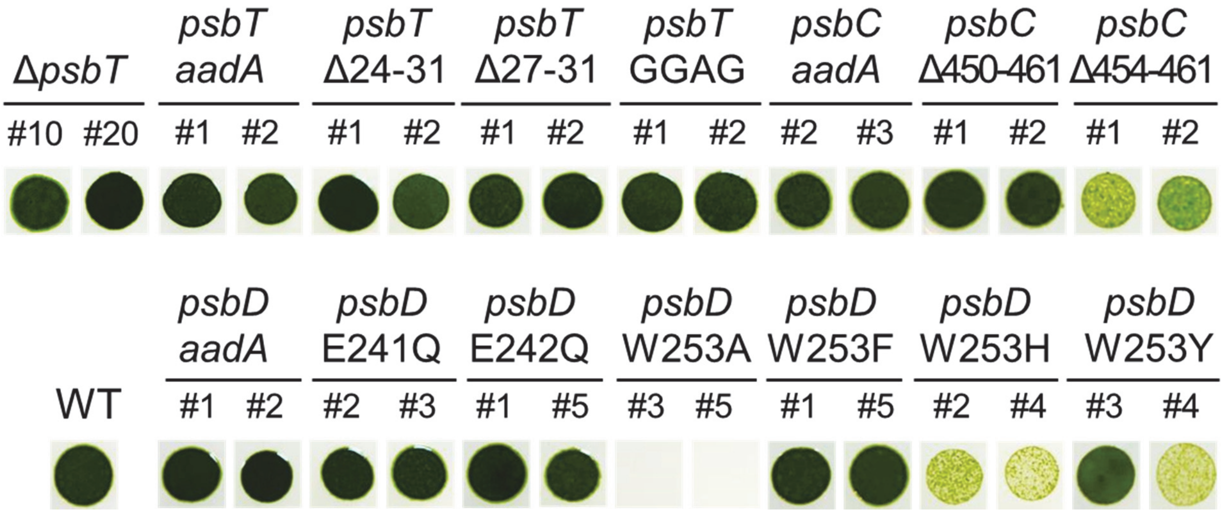

Supplementary Figure 2 | Phototrophic growth of transformed strains.

Exponentially growing cells grown in TAP medium were resuspended in water (2×10^5 cells mL^{-1}), deposited as drops on minimum medium (devoid of a source of reduced carbon) agar plates and allowed to grow for ten days under LED light (51 μmol photons m^{-2} s^{-1}). Two independent transformants are shown for each construct.

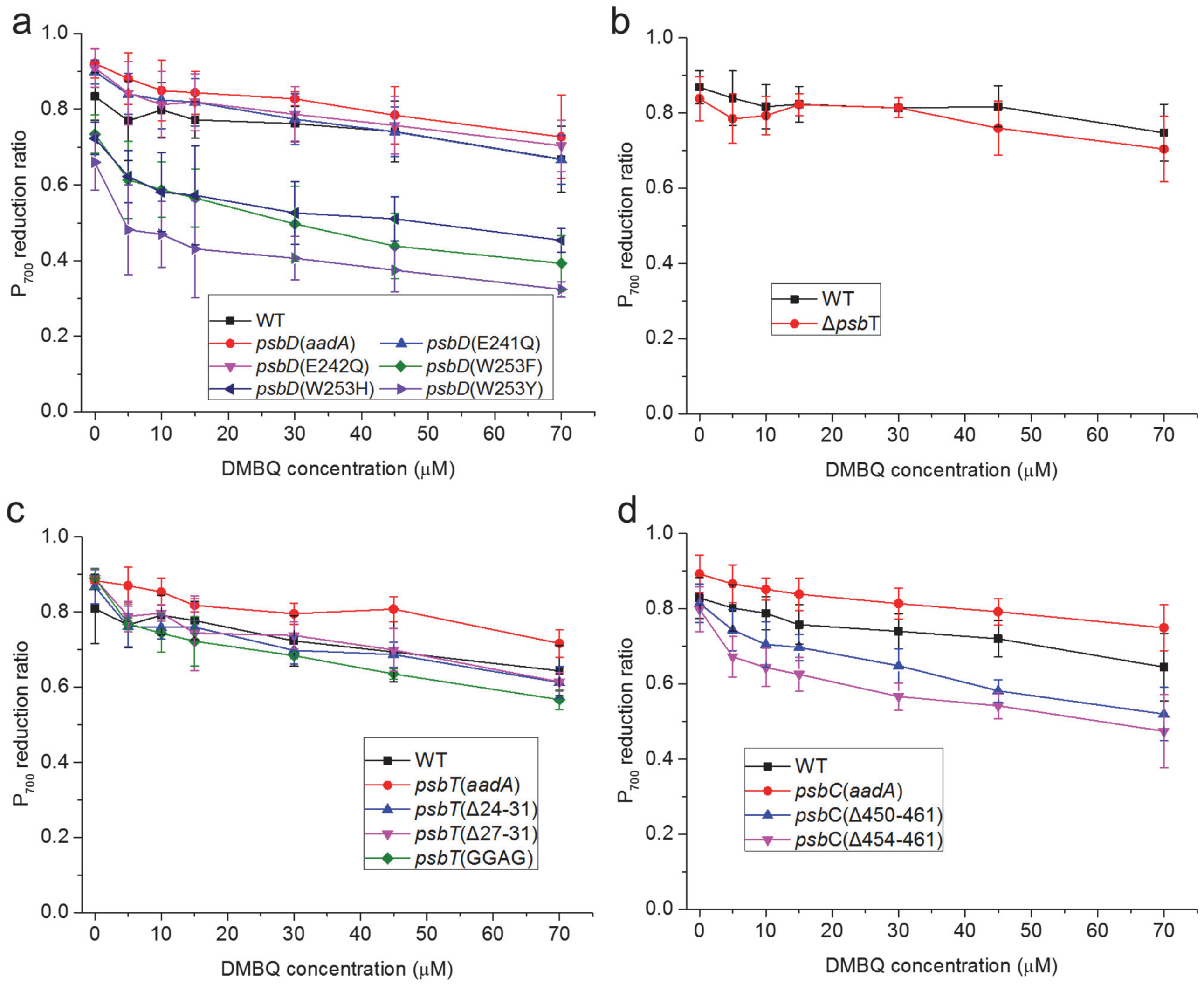

Supplementary Figure 3 | Comparison of the P_{700} reduction ratio over the DMBQ concentration in the mutant strains.

The P_{700} reduction ratio over the DMBQ concentration was estimated in the *psbD* (**a**), *psbT* deletion (**b**), *psbT*-C-terminus (**c**), and *psbC*-C-terminus (**d**) mutant strains. The data are presented as mean±s.d. of at least three independently performed experiments. The corresponding plots of the $k_{red}/k_{red(0)}$ ratio converted from the P_{700} reduction ratio are shown in Fig. 6. It is of note that the three D2-W253 mutants sustained a lower flux from PSII to PSI so that the reduction state of P_{700} remained significantly lower than in the control *psbD*(*aadA*)

strain. In addition, this flux steeply decreased with the addition of DMBQ, with a sensitivity that seemed larger than in the control strain, suggesting that the mutations open an electron transfer pathway towards exogenous electron acceptor, competing relatively efficiently with the PSII-to-PSI flow. In contrast, the E241 and E242 mutants were indistinguishable from the control strain.

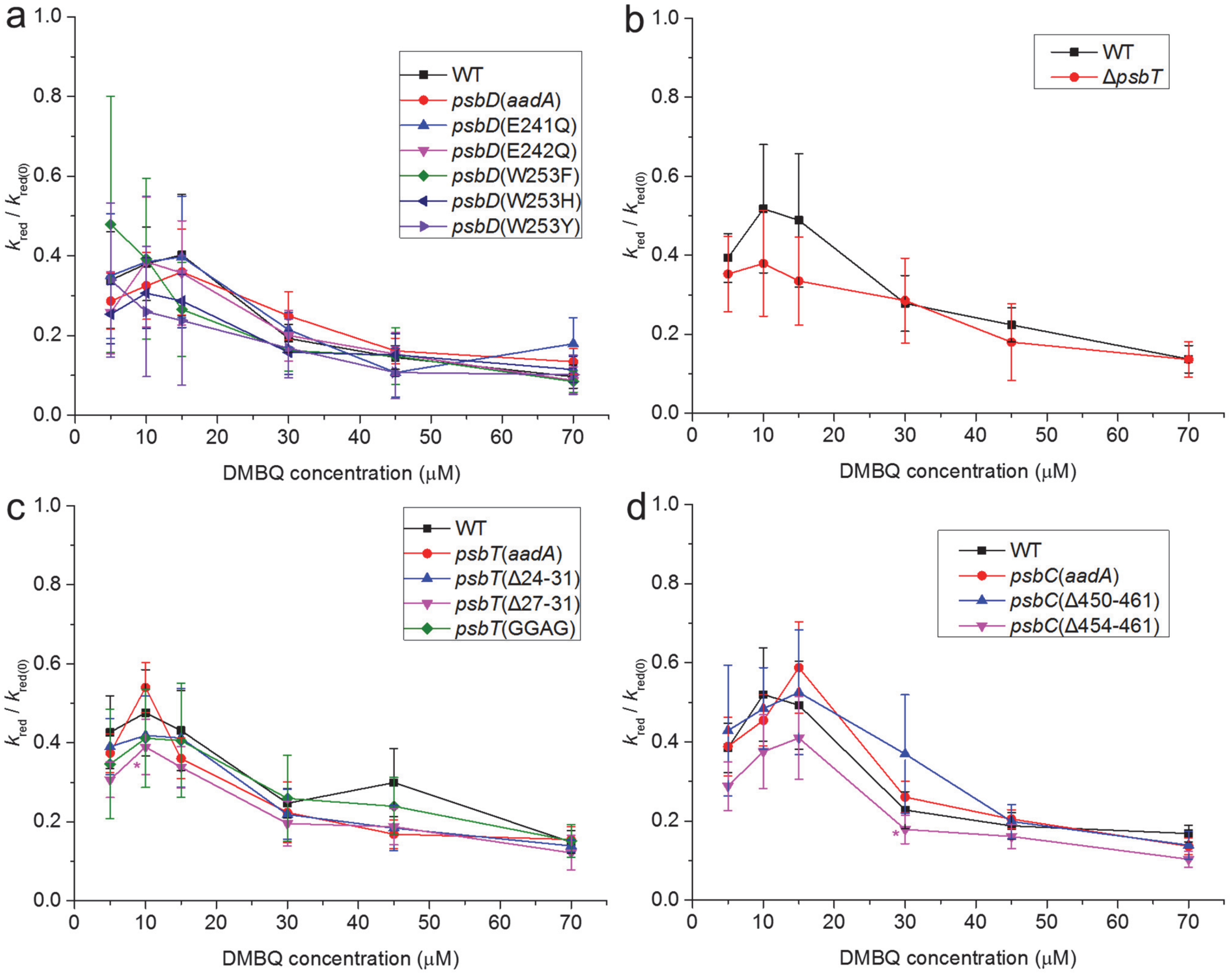

Supplementary Figure 4 | Comparison of the efficiency of electron extraction by DMBQ in the presence of DCMU in the mutant strains.

The efficiency of electron extraction was measured by the normalized $k_{red}/k_{red(0)}$ ratio determined from the P_{700} absorbance changes in the *psbD* (**a**), *psbT* deletion (**b**), *psbT*-C-terminus (**c**), and *psbC*-C-terminus (**d**) mutant strains. Asterisks indicate a statistically significant difference with respect to the corresponding *aadA* control strain or a significant difference with respect to WT for the Δ*psbT* strain using a two-tailed *t*-test. *: $0.001<P<0.05$. Error bars are s.d. of at least three independently performed experiments.

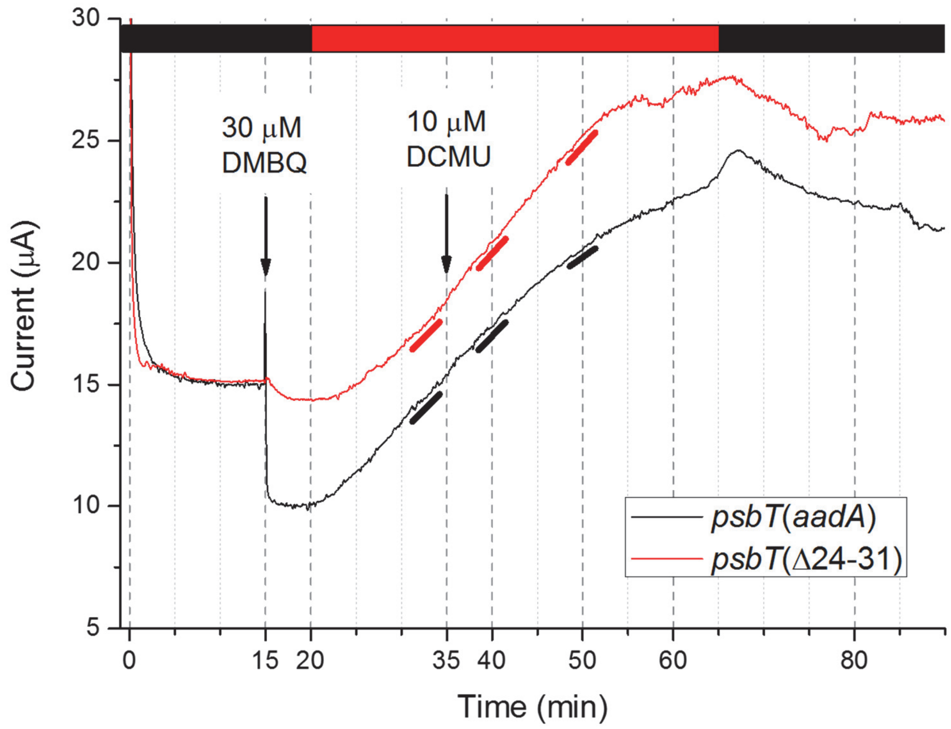

Supplementary Figure 5 | Representative chronoamperometric traces in the *psbT*(*aadA*) and *psbT*(Δ24-31) strains.

The illumination period of green LED light (30 μmol photons $m^{-2} s^{-1}$) is indicated as a horizontal red bar. Arrows represent the addition of the indicated chemicals at their final concentrations. The estimated slope of current is indicated by a bold line. See Methods for more details.

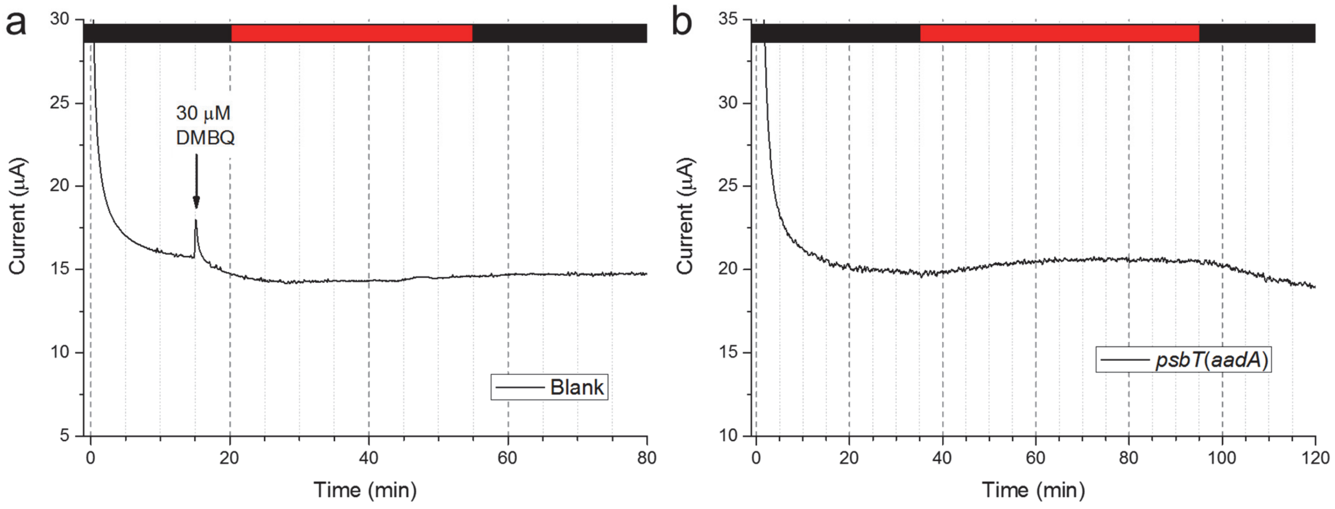

Supplementary Figure 6 | No significant current rise when either *C. reinhardtii* cells or DMBQ is absent.

Representative chronoamperometric traces in the absence of *C. reinhardtii* cells (**a**) and in the absence of DMBQ (**b**). The illumination period is indicated as a horizontal red bar; the arrow represents the addition of DMBQ at a final concentration of 30 µM.

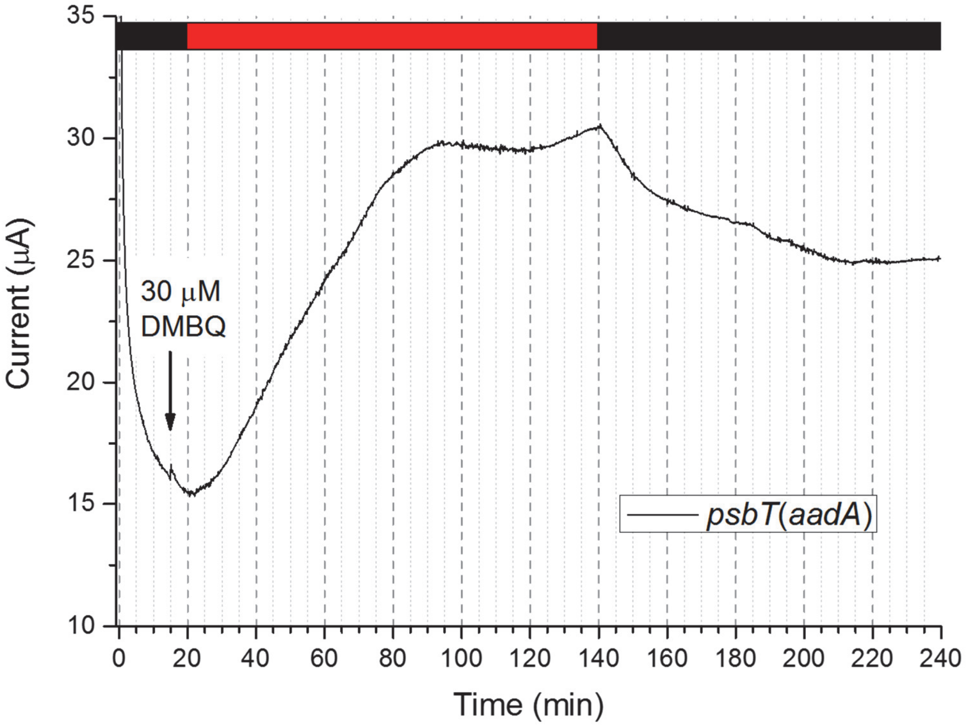

Supplementary Figure 7 | Representative chronoamperometric trace in the *psbT*(*aadA*) strain in absence of DCMU.

The illumination period is shown as a horizontal red bar; the arrow points to the addition of DMBQ at a final concentration of 30 μM.

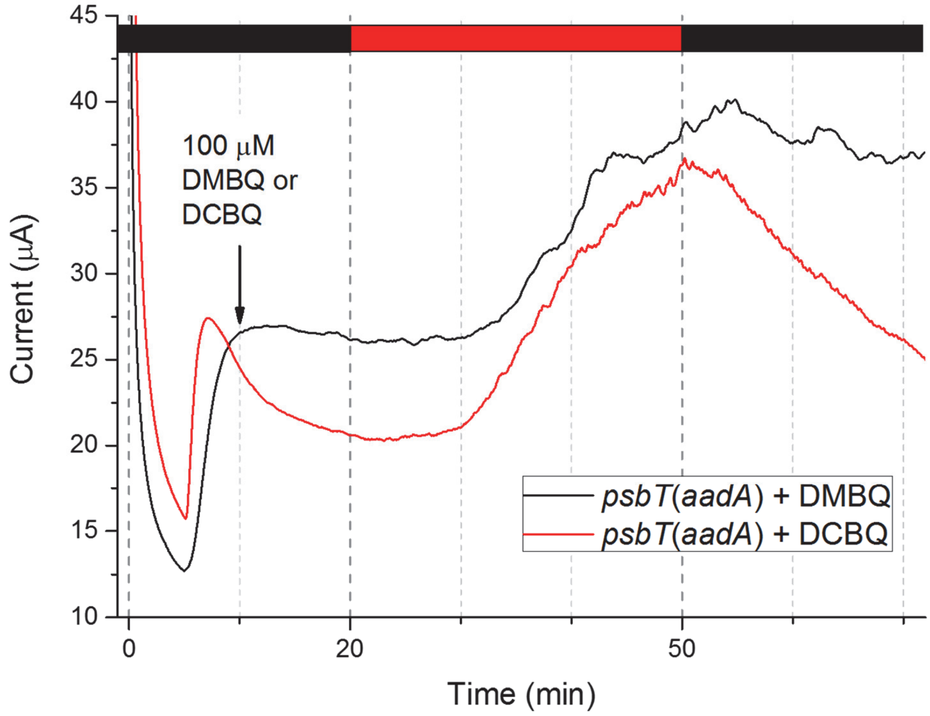

Supplementary Figure 8 | Similar levels of current rise in the *psbT*(*aadA*) strain when adding DMBQ or DCBQ.

The illumination period is shown as a horizontal red bar; the arrow points to addition of DMBQ or DCBQ at a final concentration of 100 μM.

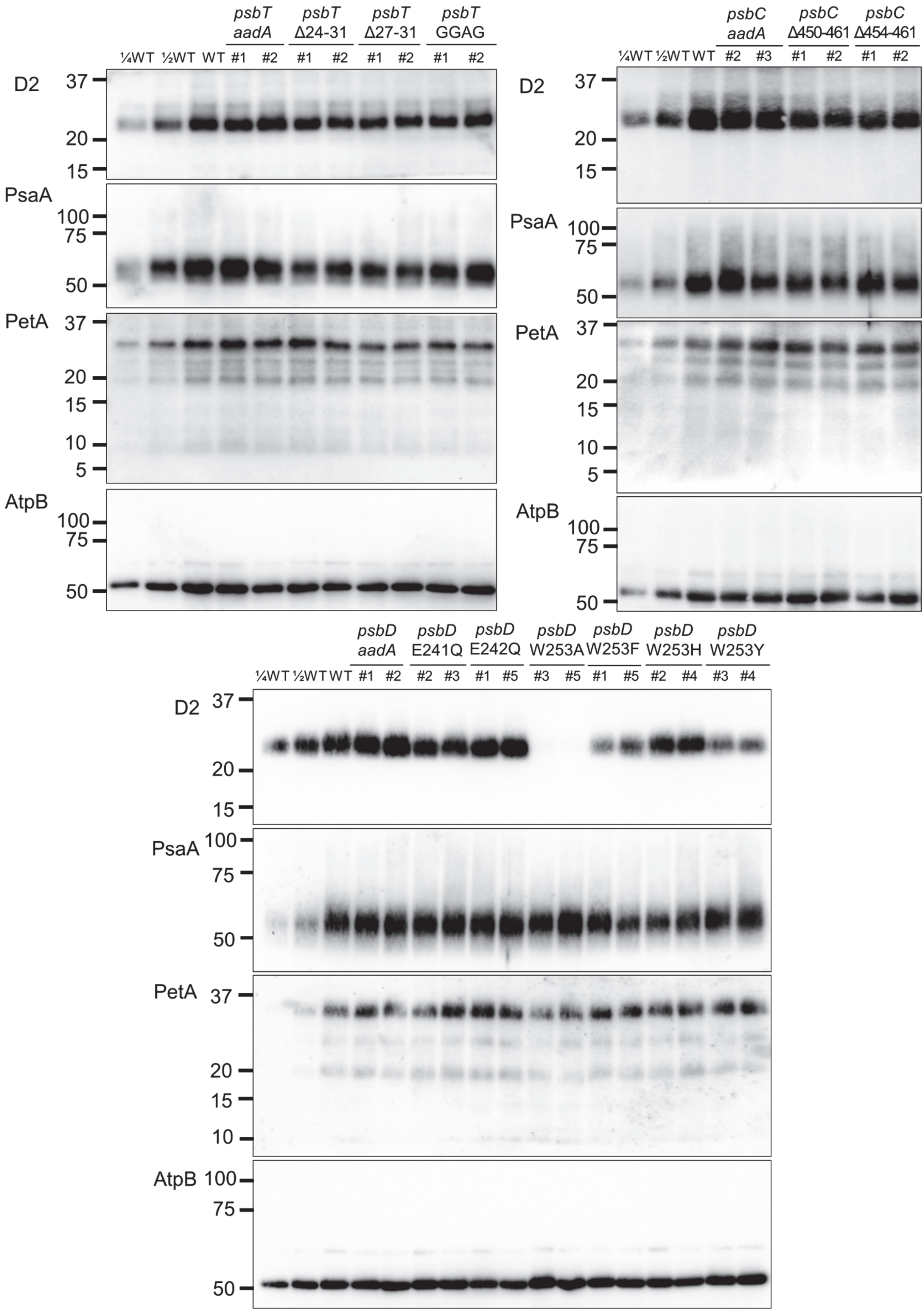

psbT aadA
psbT Δ24-31
psbT Δ27-31
psbT GGAG
¼WT ½WT WT #1 #2 #1 #2 #1 #2 #1 #2
D2
37
20
15
PsaA
100
75
50
PetA
37
20
15
10
5
AtpB
100
75
50
psbC aadA
psbC Δ450-461
psbC Δ454-461
¼WT ½WT WT #2 #3 #1 #2 #1 #2
psbD aadA
psbD E241Q
psbD E242Q
psbD W253A
psbD W253F
psbD W253H
psbD W253Y
¼WT ½WT WT #1 #2 #2 #3 #1 #5 #3 #5 #1 #5 #2 #4 #3 #4

Supplementary Figure 9 | Immunoblot analysis of photosynthetic complexes in the mutant strains.

Uncropped blot images of Figure 5. Cells were grown in TAP medium at 25 °C under LED white light (8 μmol photons $m^{-2} s^{-1}$) and harvested at the mid-log phase. Two independent lines are shown for each construct. Protein samples were loaded on an equal chlorophyll basis (0.5 μg/lane), and a dilution series of WT samples is shown for semi-quantitative comparison. Antibodies against essential subunits of PSII (D2), PSI (PsaA), cytochrome b_6f (PetA) and ATP synthase (AtpB) probed the accumulation of the respective photosynthetic complexes. Numbers on the left side of the blots are molecular weights in kD.

Supplementary Table 1 | List of oligonucleotides used in this study.

Oligonucleotides were synthesized for engineering the *aadA* cassette (**a**) or site-directed mutagenesis of *psbD* (**b**), *psbT* (**c**), or *psbC* (**d**). Nucleotides mutated either for site-directed mutagenesis or to create RFLP markers are written in lower case. Relevant stop codon are underlined, while introduced restriction sites, listed in the last column, are marked in bold or in italic.

Oligo name	DNA sequence (5'→3')	Restriction site
**a**		
**rbcLCod**	GCGCAATTGAGGCCTACTAGTTCTAGA**CTGCAG**AATTTGATACTATTGACAAACTTTAAT	**PstI**
**rbcLRev**	ACCATATGTGTATTCGGATCCTTCGAAAGATTTATTTTTCTGGCACATCGTA	
**psaACod**	GAAAAATAAAATCTTTCGAAGGATCCGAATACACATATGGTAAAAAATA	
**psaARev**	CGC**GCATGC**CCGGGCCATGGATTTCTCCTTATAATAACAATT	**SphI**
**b**		
**psbD-mut-EF**	CCCTTGTGCTTACTTTGCATTAG	
**aadA-ER**	TACAGCGCGGAGAATCTCG	
**psbD-W253A-IF**	CCGAAGATTT**GaGACg**cGAAACGGTTAGCAGTAACCATAGAG	**BsmBI**
**psbD-W253A-IR**	TTCg**cGTCtC**AAATCTTCGGTGTTGCTTTCTCTA	**BsmBI**
**psbD-W253F-IF**	TGAgaAGA**AcCGGT**TAGCAGTAACCATAGAGTATGT	**AgeI**
**psbD-W253F-IR**	GCTA**ACCGgT**TCTtcTCACAAATCTTCGGTGTTGCTTTCTCT	**AgeI**
**psbD-W253H-IF**	CGA**AGATcT**GTGAgtgGAAACGGTTAGCAGTAACCATAGAGT	**BglII**
**psbD-W253H-IR**	TTCcacTCAC**AgATCT**TCGGTGTTGCTTTCTCTAACAA	**BglII**
**psbD-W253Y-IF**	TGAgtAGA**AtCGaT**TAGCAGTAACCATAGAGTATGTTTCTTC	**ClaI**
**psbD-W253Y-IR**	GCTA**AtCGaT**TCTacTCACAAATCTTCGGTGTTGCTTTCTCT	**ClaI**
**psbD-E241Q-IF**	AGTA**gGTcTC**TTgAGCCTGTGTAGGGTTGAATGCACGG	**BsaI**
**psbD-E241Q-IR**	ACAGGCTcAA**GAgACc**TACTCTATGGTTACTGCTAACCGTTTCTG	**BsaI**
**psbD-E242Q-IF**	CATAGAGTATGTTTgT**TCcGCC**TGTGTAGGGTTGAATGCACGGAAT	**EciI**
**psbD-E242Q-IR**	CACA**GGCgGA**AcAAACATACTCTATGGTTACTGCTAACCGTTTC	**EciI**
**c**		
**psbT-aadA-EF**	TAAGGCGACGTCCTTTAGG	
**psbT-aadA-ER**	TGCATCCCTTTCAGGGTAG	
**psbT-aadA-IF**	TAAG**AtcgAT**TAA*AccggT*TATGGACCTACAGAGTTGG	**ClaI**; *AgeI*

**psbT-aadA-IR**	CATAA*AccggT*TTA**ATcgaT**CTTAAGAGGGTAGTATTTAATAG	*AgeI*; **ClaI**
**psbT-R24stop-IF**	CAATTTTCTTCta**gGATCC**TCCACGTATGATTAAAT	**BamHI**
**psbT-R24stop-IR**	GTGGA**GGATCc**taGAAGAAAATTGAGAAGAAAATAATAC	**BamHI**
**psbT-P27stop-IF**	CAGAGATCCTtgA**CaTATG**ATTAAATAATAGCATTTA	**NdeI**
**psbT-P27stop-IR**	TATTTAAT**CATAtG**TcaAGGATCTCTGAAGAAAATTGAG	**NdeI**
**psbT-GGAG-IF**	CTTCgGAG**gTgCag**gACGTATGATTAAATAATAGCATTTAA	**BsgI**
**psbT-GGAG-IR**	TACGTc**ctGcAc**CTCcGAAGAAAATTGAGAAGAAAATAATAC	**BsgI**

d

**psbC-aadA-EF**	CTCCACGTTCATGGTTAGC	
**psbC-aadA-ER**	TTGAACCGGTGACCCAAG	
**psbC-aadA-IF**	CGCT**CAatTG**A*CcCGGg*AAATGCCAATCTTTTTAATAACTTTATAG	**MfeI**; *XmaI*
**psbC-aadA-IR**	ATTT*cCCGgG*T**CAatTG**AGCGATTTCAATGGTTTGTTAGTAA	*XmaI*; **MfeI**
**psbC-F450stop-F**	CGTGCACGTGCTGCTGCTGCTGGTTTCGAAAAAGGTATCGACCGTT**gaGACG** AACCAGTTCTTTCAATGCGTCC	**BsmBI**
**psbC-V454stop-F**	CGTGCACGTGCTGCTGCTGCTGGTTTCGAAAAAGGTATCGACCGTTTCGACG AACCAta**gCaTTC**AATGCGTCCTTTAGACTAATTTT	**BsmI**
**psbC-stop-R**	CATTGCGCTGCCATTCTC	

Supplementary Table 2 | Computation of minimum distances to estimate the accessibility of PSII to DMBQ with C-terminal mutations of CP43 or PsbT.

The minimum distances were either measured between the conjugated ring of Q_A and the solvent-excluded surface of PSII (middle column), or measured between the conjugated rings of Q_A and DMBQ among 12 binding modes generated by the AutoDock Vina molecular docking program (right column). The edge-to-edge distances should be at least about 1.4 Å larger than the corresponding distances to excluded surfaces.

Strain	Minimum distance between the conjugated ring of Q_A and the solvent-excluded surface of PSII (Å)	Minimum distance between the conjugated ring of Q_A and the docked DMBQ molecule (Å)
WT	9.3	10.5
psbC(Δ450-461)	8.6	10.3
psbC(Δ454-461)	8.6	10.9
psbT(Δ24-31)	6.4	8.8
psbT(Δ27-31)	7.0	10.9
psbT(GGAG)	8.4	10.2
